# Porous MnFe_2_O_4_-decorated PB nanocomposites: a new theranostic agent for boosted *T*_1_/*T*_2_ MRI-guided synergistic photothermal/magnetic hyperthermia†

**DOI:** 10.1039/c8ra02946f

**Published:** 2018-05-22

**Authors:** Xi Zhou, Xiaolin Lv, Wen Zhao, Tiantian Zhou, Shupeng Zhang, Zhan Shi, Shefang Ye, Lei Ren, Zhiwei Chen

**Affiliations:** Key Laboratory of Biomedical Engineering of Fujian Province University/Research Center of Biomedical Engineering Technology of Xiamen, Department of Biomaterials, College of Materials, Xiamen University Xiamen 361005 P. R. China yeshefang@xmu.edu.cn; Department of Electronic Science, Fujian Provincial Key Laboratory of Plasma and Magnetic Resonance Research, Xiamen University Xiamen 361005 P. R. China chenzhiwei@xmu.edu.cn; Department of Materials Science and Engineering, College of Materials, Xiamen University Xiamen 361005 P. R. China

## Abstract

This study reports a multifunctional core/shell nanoparticle (NP) that can be used for amplified magnetic resonance image (MRI), enhanced photothermal therapy (PTT) and magnetic hyperthermia therapy (MHT) due to its surface coating with a porous shell. Importantly, by means of introducing the surface coating of a porous shell, it helps entrap large quantities of water around NPs and allow more efficient water exchange, leading to greatly improved MR contrast signals. Besides, the porous shell helps the near-infrared (NIR) absorbance of the core, and then the extremely enhanced thermal effect can be obtained under synergistic combination of PTT and MHT. By synthesizing multifunctional porous MnFe_2_O_4_/PB as an example, we found that the transversal relaxivity (*r*_2_) of MnFe_2_O_4_ NPs might improve from 112.11 to 123.46 mM^−1^ s^−1^, and the specific absorption rate (SAR) of MnFe_2_O_4_/PB nanoparticles reached unprecedented levels of up to 4800 W g^−1^ compared with the SAR 1182 W g^−1^ of PTT under an 808 nm laser and 180 W g^−1^ of MHT under an external AC magnetic field. Meanwhile, when MnFe_2_O_4_ was decorated on PB nanoparticles, the magnetic properties became lower slightly, but the synergistic photothermal/magnetic hyperthermia conversion was enhanced greatly. Subsequently, *in vitro T*_1_–*T*_2_ dual-modal MRI, PTT and MHT results verified that MnFe_2_O_4_/PB could serve as an excellent MRI/PTT/MHT theranostic agent. Furthermore, the MnFe_2_O_4_/PB NPs were applied as a *T*_1_–*T*_2_ dual-modal MRI, PTT and MHT theranostic agent for *in vivo* MRI-guided photothermal and magnetic hyperthermia ablation of tumors by intratumoral injection in 4T1 tumor-bearing mice. The *T*_1_–*T*_2_ dual-modal MR imaging result shows a significantly contrast in the tumor site. The MPB-mediated PTT and MHT result shows high therapeutic efficiency as a result of high photothermal and magnetic hyperthermia conversion efficiency. The multifunctional NPs have a great potential application for future clinical tumorous diagnosis and treatment.

## Abstract 2

We synthesized a new theranostic agent of porous MnFe_2_O_4_-decorated PB nanocomposites for boosted *T*_1_/*T*_2_ MRI-guided synergistic photothermal/magnetic hyperthermia.

## Graphical abstract

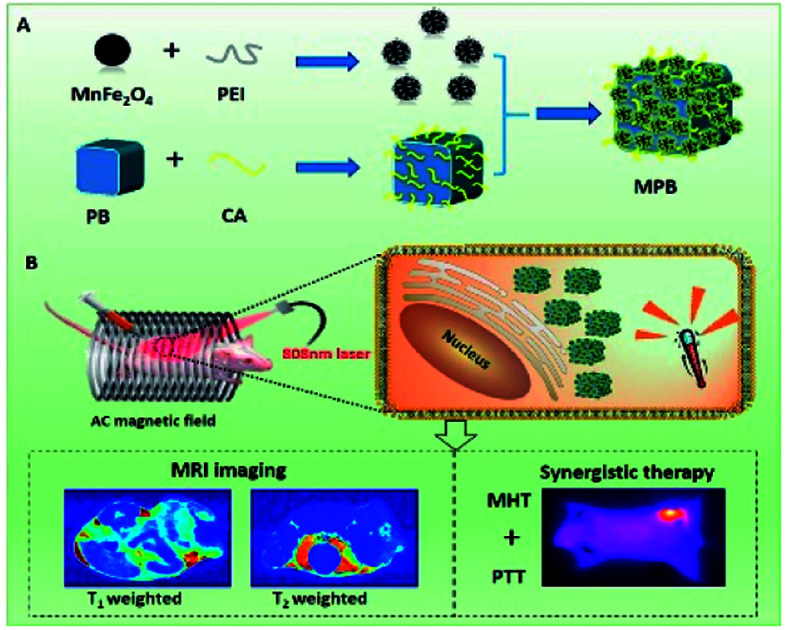

## 1. Introduction

There have been major developments in the combination of nanotechnology and cancer theranostic technology over the past few years.^1–3^ Accurate early detection of cancer is highly desirable so that appropriate therapy can be provided before the disease becomes too serious. Also, theranostic nanomaterials have enabled improved treatment efficacy of cancer through multimodal image-guided combination therapy.^4–6^ There have been a number of nanoparticles created by combining diagnostic technologies including photo-acoustic imaging (PA), magnetic resonance imaging (MRI), and X-ray computed tomography (CT) with a therapeutic approach such as radiotherapy, chemotherapy, magnetic hyperthermia (MHT), and photothermal therapy (PTT).^7–10^ This has enabled potential applications for the treatment of cancer. Among them, MRI has attracted considerable attention since it can provide images with excellent spatial resolution without ionizing radiation. However, the low signal intensity of transversal (*T*_2_)-weighted MRI may not be clearly differentiated from the surrounding tissue in some lesions. In contrast, longitudinal (*T*_1_) imaging has an excellent resolution between tissues due to its high signal intensity, hence, the *T*_1_–*T*_2_ dual-modal strategy for MRI has attracted considerable interest because it can give highly accurate diagnostic information due to its beneficial contrast effects.^11–13^ Furthermore, PTT employs NIR, light-absorbing agents to generate heat from optical energy that leads to thermal ablation of cancer cells which are more heat-sensitive than normal tissues.^14,15^ MHT can use super paramagnetic NPs to transform the AC magnetic field energy into heat due to its magnetic relaxational losses.^9^ The raised temperature of the cancer region will cause the necrosis of the cancer cells. Thus, nanocomplexes with *T*_1_/*T*_2_ MRI-guided synergistic PTT and MHT functions will significantly boost theranostic efficiency due to their intrinsic characteristics that combine high spatial resolution, noninvasiveness, and selective visualization of different tissues of *T*_1_–*T*_2_ dual model MRI, and more importantly, the extremely high efficiency of the synergistic PTT and MHT, compared with the single theranostic technique.

Core–shell multifunctional nanomaterials have been widely utilized in theranostic fields such as biomedical imaging, cancer treatment and the diagnosis of disease. For instance, Lu/Y@Lu UCNCs,^16^ Fe_3_O_4_@Au NP_S_,^17^^125^I-fSiO_4_@SPIOs^6^ and PB@Au^5^ NPs have recently been explored as optical fluorescence, MRI, SPECT and CT imaging nanoprobes. In addition, CoFe_2_O_4_@MnFe_2_O_4_/polypyrrole nanocomposites,^18^ and Ag/Fe_3_O_4_ nanoflowers^19^ have been successfully integrated into a nanoplatform to establish the combination of photothermal and magneto thermal therapy. However, core–shell multifunctional nanoparticles usually suffer from the functional shielding of the “core” by the shell, leading to low-efficiency performance of the core materials. For example, the magnetic core–shell NPs usually suffer from a poor relaxation rate and low-efficiency MRI performance when these NPs are functionalized with most types of materials (*e.g.*, mesoporous silica and gold NPs). Similarly, the optical performance of the core material will also be affected by the shell material. Until now, several strategies have been suggested to eliminate the shielding effect of the shell material to the core material. An effective method to achieve this is to coat the magnetic core NPs with a layer of functional hydrophilic polymers, which can entrap large quantities of water around the core NPs to improve the relaxation rate and simultaneously provide a variety of therapeutic capabilities. For instance, Chen *et al.* reported that the surface coating of human serum albumin (HSA) could enhance the *r*_1_ relaxivity of MnO NPs from 0.37 to 1.97 mM^−1^ s^−1^, which enables HSA–MnO NPs to perform well in MRI/PET dual imaging.^20^ Recently, our group reported that the manganese carbonate nanoparticles coated by polydopamine can dramatically improve MR contrast signals (*r*_1_ relaxivity improved from 5.70 to 8.30 mM^−1^ s^−1^) and enable cancer photothermal therapy.^21^ However, for the richly functional inorganic core–shell NPs, it remains a great challenge to acquire high-performance theranostic agents with the improvement property of the core and shell materials.

**Scheme 1:**
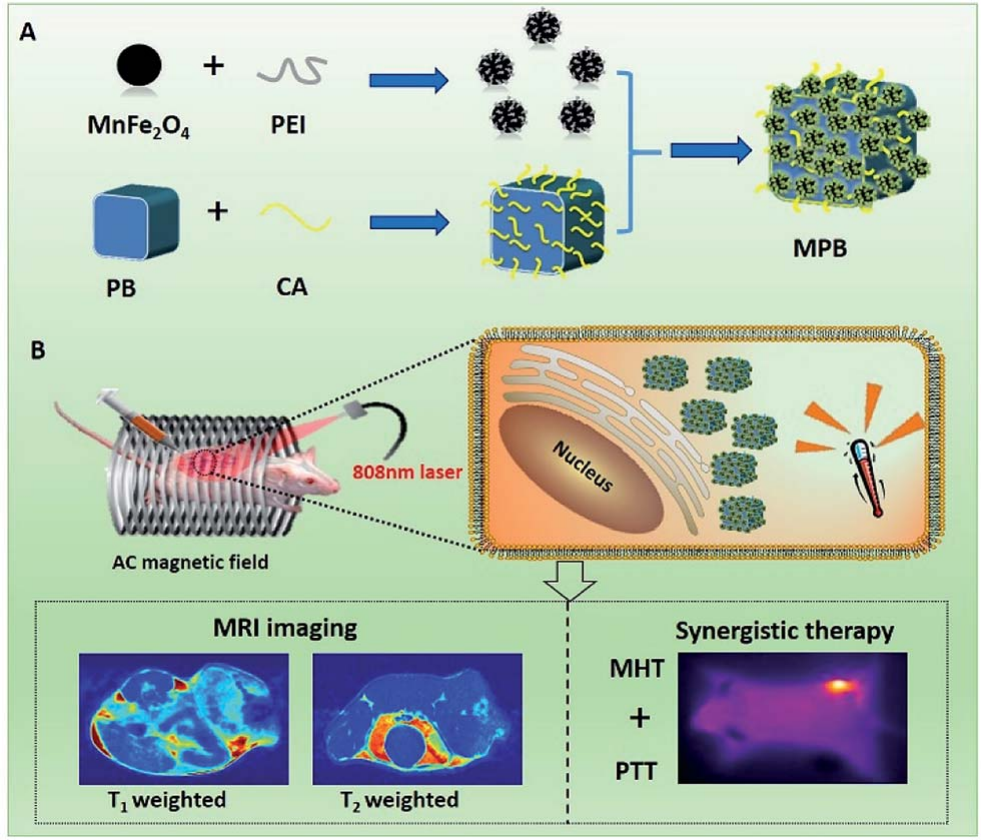
Synthesis of MPB NPs and their applications for *T*_1_/*T*_2_ MRI-guided synergistic photothermal/magnetic hyperthermia.

Prussian blue (PB)^22,23^ has emerged as the latest generation of agents for both MR imaging and photothermal tumor ablation since it has the ability to shorten the *T*_1_ relaxations of protons from bulk water and exhibit a strong light absorbance and super photothermal conversion efficiency in the near infrared region.^24–29^ Meanwhile, as important magnetic ternary nanoparticles, the MnFe_2_O_4_ nanoparticle possesses a stronger magnetization intensity value due to the smaller magnetocrystalline anisotropy and the fact that it is relatively easy to reverse magnetize, making it a potential MR imaging and magnetic hyperthermia agent.^30–32^ Here, by using a multi-functional magnetic Prussian blue nanoparicles-MnFe_2_O_4_/PB (MPB) nanocomposite as an example, we present a versatile strategy to improve the performance of core–shell nanostructures through controlling the surface structure of the shell. We have successfully fabricated a porous MnFe_2_O_4_-decorated PB nanocomposite as a potential MRI/PTT/MHT theranostic agent in Scheme 1. Benefiting from the surface coating of porous MnFe_2_O_4_ layer, the longitudinal relaxivity of PB NPs has not be impacted by the shielding effect of the MnFe_2_O_4_ layer. And the transversal relaxivity of MnFe_2_O_4_/PB becomes much higher than that of the MnFe_2_O_4_ NPs and the value of *r*_2_ improves from 112.11 mM^−1^ s^−1^ of MnFe_2_O_4_ to 123.46 mM^−1^ s^−1^ of MPB. Furthermore, the porous shell layer is good for the near-infrared light absorbance of PB, and the heated performance of the synergistic combination of PTT and MHT has been extremely enhanced. Compared with the SAR 1182 W g^−1^ of PTT under an 808 nm laser and 180 W g^−1^ of MHT under an external AC magnetic field, the SAR of MPB nanoparticles reached unprecedented levels of up to 4800 W g^−1^. Subsequently, the MPB NPs were applied as an MRI/PTT/MHT theranostic agent for the *in vivo* MRI-guided photothermal and magnetic hyperthermia ablation of tumors by intratumoral injection in murine breast cancer cells (4T1) tumor-bearing mice. This nanoformulation enabled the high-resolution tumor *T*_1_/*T*_2_ MR imaging of small animals. The MPB-mediated PTT and MHT result shows obvious therapeutic efficiency for cancer ablation and our proposed strategy suggests that these materials have the function of multi-imaging and double treatments, integrated with diagnosis and treatment. This may have a great potential application for future clinical tumorous diagnosis and treatment.

## 2. Experimental

### 2.1 Chemicals and reagents

Potassium hexacyanoferrate(iii) trihydrate (K_3_[Fe(CN)_6_]·3H_2_O), iron(iii) chloride hexahydrate (FeCl_3_·6H_2_O), citric acid (CA), polyetherimide (PEI, *M*_w_ ≈ 10^4^) and manganese chloride tetrahydrate (MnCl_2_·4H_2_O) were purchased from Sinopharm Chemical Reagent Co. Ltd. (Shanghai, China). Poly(vinylpyrrolidone) (PVP, K30) were obtained from Aladdin Industries Incorporation. Hydrochloric acid (HCl), sodium hydroxide (NaOH), ethyl alcohol and acetone were purchased from Shantou Xilong Chemical Factory (Guangdong, China). Phosphate buffer solution (PBS), Dulbecco's modified Eagle's medium (DMEM), fetal bovine serum (FBS), and 3-(4,5-dimethylthiazol-2-yl)-2,5-diphenyltetrazolium bromide (MTT) were got from Gibco. Dimethyl sulfoxide (DMSO) were purchased from Biological Industries (Beit Ahemeq, Israel). Calcein acetoxymethyl ester (Calcein AM), Annexin V-FITC and propidium iodide (PI) were purchased from Nanjing KeyGen Biotech Co. Ltd. (Nanjing, China). All chemicals were used without further purification. Ultrapure water (18.2 MΩ cm) was obtained from Milli-Q water purification system.

### 2.2 Preparation of Prussian blue nanoparticles (PB NPs)

PB NPs were prepared according to the previous literature.^33^ Typically, 1.500 g PVP was added into 20 mL H_2_O, then, 6 M HCl was added dropwisely into the above solution under stirring at 60 °C, until the pH reached to the 2. A clearly bright dispersion formed immediately during the mixing process. Then, 45 mg K_3_[Fe(CN)_6_]·3H_2_O and 192 mg CA were added into above solution, stirring for 15 min, then transferred into a Teflon-lined stainless-steel autoclave with a capacity of 50 mL. The autoclave was heated to 80 °C and maintained for 2 h, and then allowed to cool to room temperature. Finally, 25 mL acetone was added into the dispersion then centrifuged at 12 000 rpm for 12 min to collect PB NPs. Subsequently the obtained PB NPs were washed with acetone for three times and re-dispersed in water for future use.

### 2.3 Preparation of MnFe_2_O_4_ magnetic nanoparticles

MnFe_2_O_4_ nanoparticles were prepared by co-precipitation method according to the previous literature.^34^ Firstly, 0.221 g MnCl_2_·4H_2_O and 0.600 g FeCl_3_·6H_2_O were dissolved in 22.5 mL water and the mixture was stirred for 10 min under room temperature. Then, 0.750 g NaOH was added into the solution rapidly with vigorous stirring at 80 °C. Secondly the 0.050 g PEI which dispersed into 3.0 mL DI water was added into the solution, and stirred for 20 min. Materials were separated by magnetic decantation and repeatedly washed with water and ethanol. Finally, the obtained MnFe_2_O_4_ NPs were dissolved in water for future use.

### 2.4 Preparation of MPB magnetic nanoparticles

Putting proper PB solution and MnFe_2_O_4_ solution together with the concentration of PB and MnFe_2_O_4_ (*m*PB : *m*MnFe_2_O_4_ ≈ 1 : 2.3), 12 M HCl was added dropwisely into the solution until the pH reached to 3. After mechanical stirring for overnight, then NPs were separated by magnetic decantation and repeatedly washed with water and ethanol. Then, the final product was dried in an oven at 60 °C. Finally, the obtained MPB NPs were dissolved in water for future use.

### 2.5 Characterization and instrumentation

The structure and morphology of PB, MnFe_2_O_4_ and MPB were characterized by scanning electron microscopy (SEM, LEO-1530, Japan), transmission electron microscopy (TEM, JEM-1400F, Japan), and scanning transmission electron microscopy (STEM, FEI Company). The zeta-potentials and size distributions were measured by Malvern Zetasizer Nano ZS (Malvern Instruments Ltd., Worcestershire, U.K.). The phase and crystallography of the product were characterized by X-ray diffraction (XRD) equipped with Cu Kα radiation (*λ* = 1.542 nm) over the 2*θ* range of 20–60°. The UV-vis absorption spectra curves were detected by UV spectrophotometer (TU-1810). Optical fiber thermometer was used to record the change of the temperature. High frequency heating equipment (10-SPG10AB) was purchased from the Shuangping high-frequency heater factory of Shenzhen. The temperature change pictures of samples were collected by the infrared thermal camera, the Fourier transform infrared (FTIR) spectra were recorded on a Nicolet iS10 (Thermo Scientific, USA) with KBr pellet technique. Magnetic property characterization was performed with a vibrating sample magnetometer (VSM) on a model 20 000 physical property measurement system (Quantum) at 300 K. The concentrations of Fe^3+^ and Mn^2+^ element were measured by inductively coupled plasma-mass spectrometry (ICP-MS). The absorbance of purple formazan was measured at 490 nm using a spectrophotometric microplate reader (BioRad 680, Hercules, CA). *T*_1_ and *T*_2_ relaxation time and *in vivo* MR images measurements were recorded at 25 °C using the 7.0T Agilent small animal MRI scanner (Agilent Technologies Inc., USA).

### 2.6 NIR heating effect of PB, MnFe_2_O_4_ and MPB in dispersion

In order to examine the photothermal property of materials, the photothermal experiment was did. The solution was irradiated by 808 nm laser light at 1.0 W cm^−2^, and recorded the temperature change with the infrared thermal camera. The laser power density was calculated by the following equation:*η* = *P*/π*R*^2^,where the *η* is laser power density, W cm^−2^; the *P* is the laser intensity, W; and the *R* is the radius of laser spot, cm. Then, vary concentrations of MPB were texted, and the experiment of thermal stability was arranged because the MPB would be used as a photosensitizer.

### 2.7 Magnetocaloric effect

The particles can transform the energy of the AC magnetic field into heat by physical mechanism, the magnetocaloric effect of MPB NPs were texted, we used the optical fiber thermometer and infrared thermal camera to record the temperature changes. The heating ability of the magnetic material was calculated by specific absorption rate (SAR) in the presence of the AC magnetic field,^35^ which was defined as the power of heating magnetic material per unit g. The SAR was determined by the initial liner increase in temperature (d*T*) and per time (d*t*), just as follows:
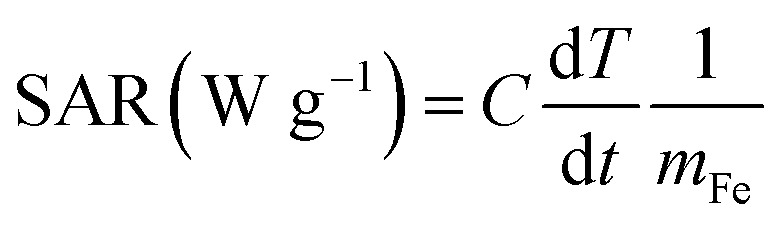
where the *C* is the specific heat capacity of the liquid solvent, the solvent in our experiment is water which can be recognized of *C*_water_ = 4185 J L^−1^ K^−1^, and *m*_Fe_ is the iron content per unit mass of the materials solutions.

### 2.8 MR contrast measurements

The as-prepared MPB NPs with different iron concentrations were dispersed in 1% agarose solution. Carr–Purcell–Meiboom–Gill (CPMG) sequence was used to measure *T*_2_ while inversion recovery (IR) sequence was used to measure *T*_1_. Common scan parameters were as follows. Repetition time *T*_R_ = 6000 ms, echo time *T*_E_ = 10, 20, 40, 80, 160, 320, 640 ms for the CPMG sequence; *T*_R_ = 9000 ms, echo time *T*_E_ = 100, 200, 400, 800, 1600, 3200, 6400 ms for the IR sequence. *T*_1_ and *T*_2_ relaxation rates were plotted against the Fe concentrations and the relaxivity was determined by a linear fit.

### 2.9 Cell and animal models

4T1 and human cervical carcinoma cells (Hela) were originally obtained from American Type Culture Collection (ATCC) and cultured under recommended medium (DMEM/1640 supplemented with 1% penicillin/streptomycin and 10% FBS) at 37 °C within 5% CO_2_ atmosphere. Female BALB/c mice were obtained from Beijing Vital River Laboratories Animal Technology Co. Ltd, and used under protocols approved by the Institutional Animal Care and Use Committee of Xiamen University. All animal procedures were performed in accordance with the Guidelines for Care and Use of Laboratory Animals of Xiamen University and approved by the Animal Ethics Committee of Xiamen University. The 4T1 tumor models were obtained by subcutaneous injection of 2 × 10^6^ cells into the right abdomen of each female BALB/c mouse.

### 2.10 *In vitro* cytotoxicity assay

*In vitro* cytotoxicity assay of MPB NPs was carried out by using the standard MTT assay on 4T1 and Hela cells. Briefly, the cells were seeded into a 96-well plate with a density of 10^4^ cells per well and cultured in the incubator with 5% CO_2_ at 37 °C for 24 h. Then, 200 μL of MPB dispersion (0, 25, 50, 100, 200, 400 μg mL^−1^) was added into the individual well and incubated at 37 °C for another 24 h. After that, cells were washed by PBS three times, then the fresh medium with 20 μL of MTT (5 mg mL^−1^) solution was added and incubated for another 4 h. Then, 200 μL of DMSO was added into the plate to dissolve the MTT formation with slight vibration for 30 min. The plate was read at 490 nm using a spectrophotometric microplate reader. Mean and standard deviations for the triplicate wells for each sample were reported.

### 2.11 *In vitro* PTT & MHT effect

The PTT & MHT effect of the MPB NPs was determined by a standard MTT assay. Firstly, for MTT assay, 4T1 and Hela cells were seeded in 96-well plates at a density of 1 × 10^4^ cells per well and cultured at 37 °C for 12 h. Then, the cells were incubated with different grade concentrations of NPs for another 4 h before being exposed to an 808 nm laser (1.0 W cm^2^, 2 min) or external alternating magnetic field (500 kHz, 12 kA m^−1^, 2 min), and the synergistic effect was obtained by operating two treatments above together. Afterward, the standard MTT assay was carried out to evaluate the cell viability. To quantify the rates of apoptosis and necrosis after *in vitro* PTT, MHT and synergistic effect, a flow cytometry of Annexin V-FITC and PI co staining was measured. Briefly, the 4T1 and Hela cells were seeded in 6-well plates and were treated with PBS, MPB + MHT, MPB + PTT and MPB + MHT & PTT respectively, and then the cells were stained with Annexin V-FITC/PI for 20 min. Subsequently, the cells were collected for flow cytometry measurement.

### 2.12 Cellular uptake

To evaluate the cellular uptake of the MPB NPs on the 4T1 and Hela cells, the cells were seeded into a 6-well plate with a density of 1 × 10^6^ cells per well and cultured in an incubator with 5% CO_2_ at 37 °C for 12 h. Then, the MPB NPs with the same concentration of 200 μg mL^−1^ were added into the plate at different time intervals (0, 1, 2, 4, 8 and 12 h), after incubation, the cells were washed three times with PBS. At last, the amounts of cell uptake were measured by ICP-MS.

4T1 and Hela cells were incubated with PBS and MPB-FITC (200 μg mL^−1^) at 37 °C for 4 h, and washed with cold PBS, and then stained with 4′,6-diamidino-2-phenylindole (DAPI) for 30 min. After that, they were fixed for 20 min in methanol and finally observed using Confocal Laser Scanning Microscope (CLSM, TCS SP5, Leika, Germany).

### 2.13 *In vivo* MRI imaging

*In vivo* MR imaging was performed on an anesthetized healthy BALB/c white mouse bearing 4T1 tumors after intratumoral injection of 50 μL MPB NPs (200 μg mL^−1^) using fast spin echo sequence *T*_1_-weighted imaging: *T*_R_ = 500 ms, *T*_E_ = 20 ms, and *T*_2_-weighted imaging: *T*_R_ = 2000 ms, *T*_E_ = 500 ms. Then the common scan parameters were as followed: FOV = 40 × 40 mm^2^, slice thickness = 1.5 mm, matrix size = 256 × 256 for all experiments. The MR images were obtained at preinjection and after intratumoral injection of the probes at preinjection, after injection 0.2, 1, 2 and 3 h.

### 2.14 *In vivo* thermal treatment

After the tumor reached 120 mm^3^ (the tumor volume was calculated by the formula of *V* = *L* × *W*^2^/2, where *V*, *L* and *W* represent the volume, length and width of the tumor respectively), the BALB/c mice were randomly divided into four groups (*n* = 3 per group), the PBS, MPB + MFH, MPB + PTT and MPB + MFH + PTT, respectively. Then, 50 μL of MPB NPs (200 μg mL^−1^) was intratumorally injected into the tumor, then the mice were irradiated with 808 nm laser light at 1.0 W cm^−2^ for 2 min, put it into an external AC magnetic field (500 kHz, 12 kA m^−1^) for 2 min respectively. On the other hand, the group of MPB + MHT + PTT was both exposed under two conditions at the same time. During the treatment, an IR thermal camera was used to obtain pictures and record the temperatures of all mice at different time intervals. The tumor sizes and body weights were measured every two days.

### 2.15 *In vivo* toxicity

At the 15th day, we euthanized the mice, and collected the major organs (brain, heart, liver, spleen, lung, and kidney) and fixed in 10% formalin for 72 h. Then, we used the paraffin to embed tissues and cryosectioned into 5 μm slices, soon after stained with hematoxylin and eosin (H&E) according to standard clinical pathology protocols. In the end we examined the stained sections with a light microscope by two pathologists, respectively.

## 3. Result and discussion

### 3.1 Synthesis and structure characterization of core/shell MPB nanoparticles

**Fig. 1:**
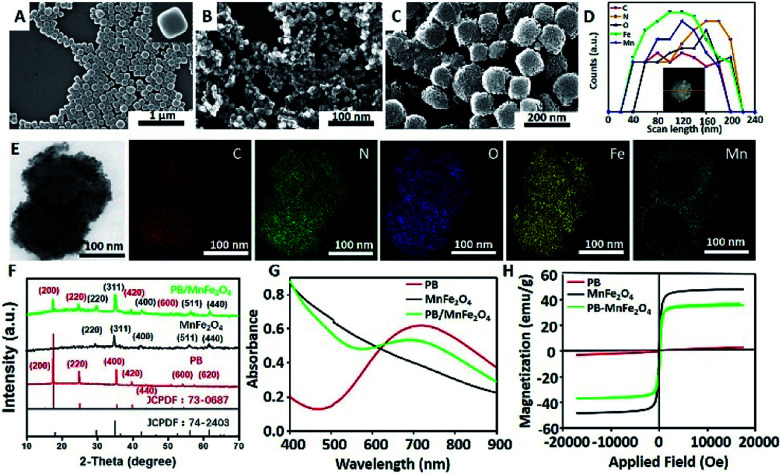
Characterizations of MPB NPs. SEM images of (A) PB, (B) MnFe_2_O_4_ and (C) MPB; (D) line-profile elemental analysis of NMPB; (E) STEM and energy-dispersive X-ray (EDX) spectroscopy elemental mapping images of MPB, indicating the presence of C, N, O, Fe and Mn elements in the nanostructure; (F) XRD patterns of PB, MnFe_2_O_4_ and MPB; (G) UV-vis absorption spectra of PB, MnFe_2_O_4_ and MPB. (H) VSM of PB, MnFe_2_O_4_ and MPB.

SEM was employed to examine the morphology of the as-synthesized MPB nanoparticles during different stages. As shown in Fig. 1A, the PB nanoparticles exhibited cubic morphology with relatively smooth surface and diameters of ∼100 nm. The zeta-potential of the PB NPs was measured at −21.3 mV (ESI, Fig. S1A†), which could be attributed to CA on the surface of the particles. Fig. 1B shown that the MnFe_2_O_4_ nanoparticles were uniform spherical nanoparticles with an average diameter of ∼20 nm, and the measured zeta-potential was +30 mV (ESI, Fig. S1†), originating from the PEI layer on the surface. This suggests that the uniform coating of CA on PB NPs and PEI on MnFe_2_O_4_ NPs benefitting the deposition of positive-charged MnFe_2_O_4_ onto the surface of negative-charged PB through the electrostatic interaction. After coating the PB nanoparticles with MnFe_2_O_4_ nanoparticles, the SEM (Fig. 1C) image shows that many small MnFe_2_O_4_ nanoparticles are adhered to the surface of the PB NPs, forming a porous shell. The porous can be seen in the Fig. 1C, and the average diameter of porous was measured to be 10–11 nm (ESI, Fig. S1B†). To further confirm the existence of the porous MnFe_2_O_4_ structure onto the PB NPs, the MPB nanoparticles were characterized by STEM analyses (Fig. 1D and E). It could be clearly observed that the elements Fe, C, O, and N were distributed uniformly in the whole MPB nanoparticle, while the element Mn mainly existed on the shell, indicating that MnFe_2_O_4_ has bound up on the surface of PB. The size distribution was measured by dynamic light scattering (DLS). We could find that the mean hydrodynamic radius of the MnFe_2_O_4,_ PB and MPB (ESI, Fig. S2A†) were 34 nm, 126 nm and 168 nm respectively (ESI, Fig. S2B†). Then, the size distributions of MPB NPs in water (ESI, Fig. S2C†) and PBS (ESI, Fig. S2D†) with different time intervals were measured by DLS, and it indicated the stability of MPB NPs. This attributed to the hydration of nanoparticles which had an excellent stability by the surface modification.^36–38^ Power XRD tests were employed to confirm the crystalline structure of the as-prepared nanoparticles. Fig. 1F showed that all peaks of the PB and MnFe_2_O_4_ NPs matched well with the pure rhombohedral phase of PB (JCPDS no. 73-0687) and MnFe_2_O_4_ (JCPDS no. 74-2403) respectively. The XRD patterns of MPB nanoparticles showed excellent agreement with the theoretical ones of PB and MnFe_2_O_4_, which further confirmed that MnFe_2_O_4_ had attached onto the PB NPs successfully. The UV-vis absorption spectra (Fig. 1G) of MPB NPs exhibited broad absorption from 600 to 900 nm, similar to the absorption properties of the free PB solution, which illustrated the structure of porous shell had no influence on the absorption of near-infrared light and ensured that the as-prepared MPB NPs could be used as NIR light-mediated thermal agent to convert the light into heat energy. In addition, in the Fig. 1H we demonstrated the saturation magnetization (*M*_s_) of the MPB NPs was 35 emu g^−1^. The magnetic property and broad absorption in NIR field of the MPB NPs suggested that it might be used in magnetic targeting, photothermal and magnetic hyperthermia during clinical trials.

### 3.2 Magnetic relaxation properties of the PB–MnFe_2_O_4_ NPs

**Fig. 2:**
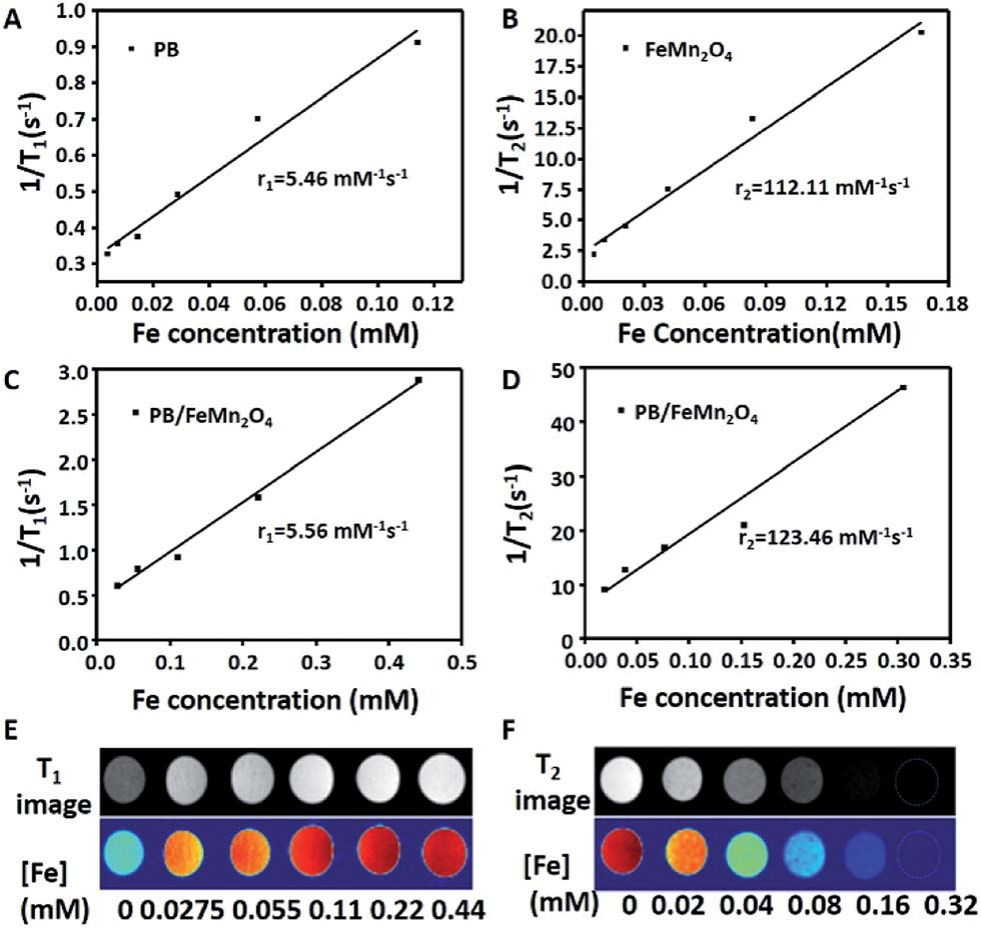
*T*_1_ and *T*_2_ contrast effects. Corresponding *T*_1_ relaxation rate of the PB (A) and *T*_2_ relaxation rate of the MnFe_2_O_4_ (B) as a function of Fe concentration. Linear relationship of *r*_1_ (1/*T*_1_) (C) and *r*_2_ (1/*T*_2_) (D) *vs.* Fe concentration of MPB. (E and F) *T*_1_- and *T*_2_-weighted MR images of the MPB NPs in aqueous solution at different Fe concentrations.

In order to evaluate the ability of the MPB nanoparticles as MR contrast agent, we investigated its magnetic relaxation properties. The *T*_1_–*T*_2_ dual mode magnetic relaxation measurements were performed using a 7.0 T small animal MRI scanner under different metal concentrations. As shown in Fig. 2A–D, we found the *r*_1_ and *r*_2_ value of MPB NPs had a slightly enhancement compared with PB and MnFe_2_O_4_ respectively. The *r*_1_ values of MPB and PB nanoparticles were 5.56 mM^−1^ s^−1^ and 5.46 mM^−1^ s^−1^, respectively; the *r*_2_ values of MPB and MnFe_2_O_4_ nanoparticles were 123.46 mM^−1^ s^−1^ and 112.11 mM^−1^ s^−1^, respectively. Those results indicated that the porous layer avoided the shielding effect of the shell, and the porous MnFe_2_O_4_ NPs on the surfaces of PB NPs avoid the spin coupling between *T*_2_ contrast materials and *T*_1_ contrast materials.^12^ Notably, these results demonstrated the versatile NPs had the capability to complete the *T*_1_ and *T*_2_ dual-modal MR imaging of MPB nanoparticles and motivated us to examine the application of MPB nanoparticles as novel contrast agents for *in vivo* MRI. The imaging spots became brighter with the increase of Fe concentration from 0 to 0.44 mM (Fig. 2E), indicated the significant increase of the *T*_1_ signal intensity, these results suggested that MPB could be an efficient *T*_1_ contrast agent. In Fig. 2F, the imaging spots became darker with the increase of Fe concentration from 0 to 0.32 mM, which indicated the NPs were excellent *T*_2_ contrast agents. All these results demonstrated that MPB composite nanoparticles were perfect *T*_1_–*T*_2_ dual-modal contrast agents and would be a promising MR contrast agent for MRI.

### 3.3 Photothermal and magnetic hyperthermia properties of NPs

**Fig. 3:**
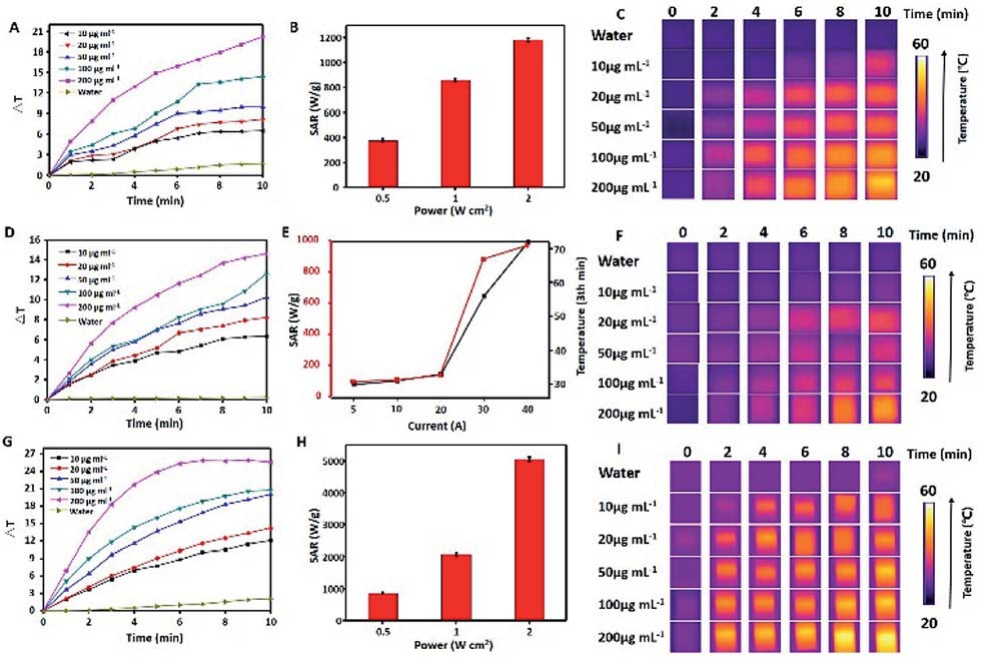
Photothermal and magnetic hyperthermia performance of MPB NPs. The temperature change curves of solutions with water and different MPB NPs concentrations after 808 nm NIR laser irradiation (A) and AC field (500 kHz, 12 kA m^−1^) (D); SAR *vs.* laser power density (B) and AC magnetic field (E) curves obtained from 0.2 mg mL^−1^ MPB NPs, in image (E) the black curve illustrated the temperature change of MPB after 3 min. IR thermal images of the water and MPB NPs suspensions at various concentrations upon NIR irradiation (C) and AC magnetic field (500 kHz, 12 kA m^−1^) (F). The temperature change curves (G), SAR *vs.* laser power density and AC magnetic field (H) and IR thermal images (I) under the dual-modal heating effect.

To evaluate the photothermal properties of MPB NPs, their aqueous solutions with different concentrations were exposed to a NIR laser (808 nm, 1.0 W cm^−2^), and the temperature of solutions were monitored. As shown in Fig. 3A, the temperature change (Δ*T*) of MPB NPs reached up to 8 °C in 3.5 min and 20 °C in 10 min at the concentration of 200 μg mL^−1^, which demonstrated NIR light-induced thermal effect of the MPB NPs. Fig. 3B shows the corresponding SAR increased from 378 to 1182 W g^−1^ with different laser powers ranging from 0.5 to 2 W cm^−2^, which indicated the great heating capacity of MPB. Then the infrared thermal images with different concentrations (0.1–0.3 mg mL^−1^) of the MPB NPs under continuous irradiation by an 808 nm laser for 10 min exhibited in Fig. 3C. Meanwhile, in the MHT-mode, the Δ*T* of the MPB solution could reach up to 7.5 °C in 3.5 min and 15 °C in 10 min with a magnetic field amplitude of 20 A (Fig. 3D). The heating power SAR increased with the amplitude and frequency of the magnetic field (best efficiency recorded at 40 A), as summarized in the red line in Fig. 3E. The black curve from Fig. 3E indicated that the temperature of MPB solutions with different AC magnetic field after 3 min. Meanwhile, Fig. 3F shows the infrared thermal images with different concentrations. It was noteworthy that the heating effect was extremely enhanced after the synergetic combination of PTT and MHT. As shown in Fig. 3G, the Δ*T* of the MPB NPs irradiated by an 808 nm NIR laser (1.0 W cm^−2^) under a magnetic field (500 kHz, 12 kA m^−1^) could be elevated by ∼20 °C in 3.5 min and ∼26 °C in 10 min, which was much higher than that of the single PTT or MHT mode. The SAR values (Fig. 3H) were even more noticeable and increased dramatically from 850 to 4800 W g^−1^ when changing the power from 0.5 W cm^−2^ to 2.0 W cm^−2^, which indicated the NPs could effectively convert near-infrared light and magnetism into thermal power. Obviously, this SAR value was much larger than that of the sum of NIR-laser and magnetic effect under the same power, which could be explained by the soft interaction between PB and porous MnFe_2_O_4_. Such efficient thermal conversion properties of the MPB NPs also provided high contrast for IR imaging (Fig. 3I). Finally, we tested the photostability of MPB solutions, and all final temperatures did not exceed 60 °C after three cycles (ESI, Fig. S3†). Therefore, the MPB NPs with efficiently synergetic photothermal & magnetic hyperthermia effect and good photostability are superior candidate for cancer thermal treatment.

### 3.4 *In vitro* cytotoxicity and uptake of cancer cells

**Fig. 4:**
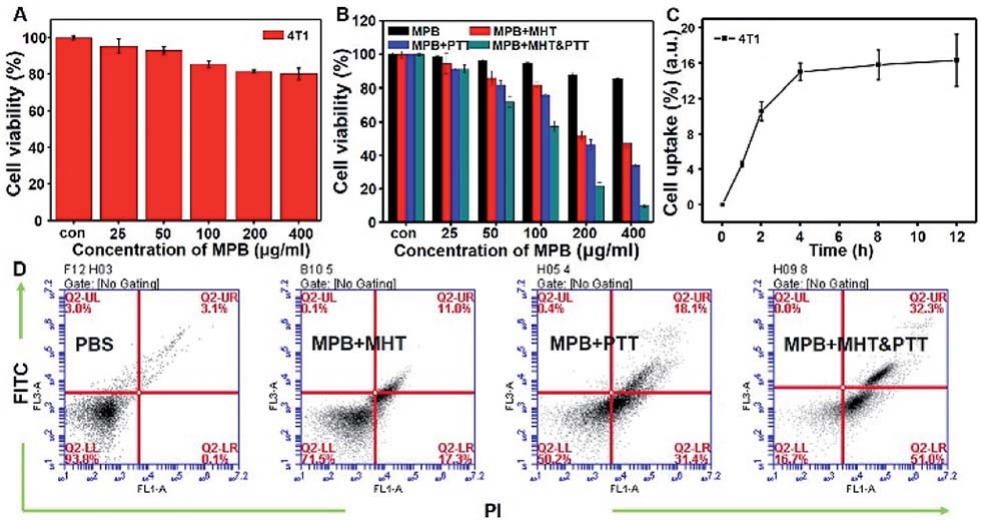
*In vitro* experiments. (A) *In vitro* cytotoxicity of MPB NPs against 4T1 cells after 24 h incubation. (B) Cell viability of 4T1 cells incubated with MPB, MPB + PTT, MPB + MHT and MPB + MHT & PTT. (C) Cellular uptake of MPB NPs at different time intervals with 4T1 cells. (D) Flow cytometry analysis the toxicities of PBS, MPB + MHT, MPB + PTT and MPB + MHT & PTT with 4T1 cells.

Before exploring the *in vivo* performance, we evaluated the biocompatibility of MPB NPs using the standard 3-(4,5-dimethylthiazol-2-yl)-2,5-diphenyltetrazolium bromide (MTT) assay. The MPB NPs exhibited negligible toxicity to 4T1 (Fig. 4A) and Hela cells (ESI, Fig. S4A†) even at a high concentration (400 μg mL^−1^) after incubation together for 24 h. However, when the 808 nm NIR irradiation (1.0 W cm^2^, 2 min) and the magnetic field (500 kHz, 12 kA m^−1^, 2 min) were applied respectively, the viability decreased significantly as the sample concentrations increased with 4T1 (Fig. 4B) and Hela cells (ESI, Fig. S4B†). As expected, the MTT result showed the cell viability under the dual-modal treatment was decreased more obviously than that with 808 nm NIR irradiation or under magnetic field. The mechanism of photothermal/magnetic hyperthermia *in vitro* is that the thermal treatment utilizing heat generated from MPB NPs under AC magnetic and NIR would ablate cancerous cells, which normally being more heat-sensitive than the normal cells. The photothermal/magnetic hyperthermia enjoys such capabilities of noninvasiveness, low number of side effect, high efficient and selectivity. Additionally, the intracellular uptake of MPB NPs was also investigated by increasing the incubation time from 0 to 12 h. During the 12 h of incubation, the uptake after 4 h showed no obvious change, demonstrating that the highest cellular uptake of the MPB NPs occurred at 4 h after incubation with the 4T1 (Fig. 4C) and Hela cells (ESI, Fig. S4C†). Then we used the FITC to label the MPB NPs, after the Hela cells were incubated with MPB NPs for 4 h, the green fluorescent signal from the endocytosis of the MPB-FITC NPs gradually increased under CLSM observation (ESI, Fig. S5†). To demonstrated the efficiency of the *in vitro* synergistic treatment, the cells were incubated with MPB and then treated under PTT, MHT or MHT & PTT modes respectively. Afterwards, they were examined by flow cytometry with the dual fluorescence of Annexin V-FITC/PI after. The population of apoptosis for 4T1 cells (Fig. 4D) and Hela cells (ESI, Fig. S4D†) treated with MPB under the dual-modal treatment was much higher than the control group of PBS and single treatment (PTT or MHT), which exhibited the excellent *in vitro* synergistic therapy efficiency.

### 3.5 *In vivo T*_1_/*T*_2_ dual-mode imaging

**Fig. 5:**
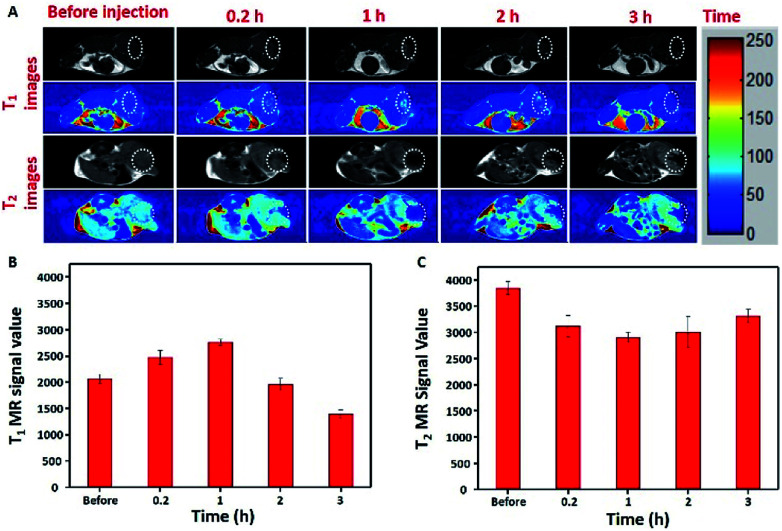
*In vivo* MR experiments of MPB NPs. (A) *T*_1_- and *T*_2_-weighted MR imaging of a 4T1 tumor-bearing mouse at different time intervals after the intratumoral injection of MPB NPs, below are the respective color coded images, the white circles indicate tumor issues. Corresponding normalized signal intensity of (B) *T*_1_-and (C) *T*_2_-weighted MR signals from the tumor at different times.

To further verify the *T*_1_–*T*_2_ dual-modal *in vivo* MR imaging, a 4T1 xenograft tumor-bearing mice were used to collect MR signals with a 7.0 T MRI scanner. As shown in Fig. 5A, *T*_1_-weighted MR images exhibited significantly brighter images at the tumor site, and the images further brighten gradually with time goes on. On the other hand, *T*_2_-weighted MR images exhibited a significantly darker images with time goes on. Similarly, it could be observed that during the time of post injection, the relative *T*_1_-weighted MRI signal value at the tumor site also increased and reached a platform at about 1 h (Fig. 5B), and *T*_2_-weighted MRI signal value had an obvious decrease within 1 h (Fig. 5C), then the value of *T*_1_ became slightly lower and *T*_2_ became higher over time which may attributed to the dissolving of MPB in the tumor environment.^39–41^ These results indicate that MPB nanoparticles have the unparalleled ability to simultaneously show strong MR contrast enhancement both in *T*_1_ and *T*_2_ imaging *in vivo*, which is able to give higher diagnostic accuracy and offer more comprehensive imaging information.

### 3.6 Dual heating *in vivo*: complete tumor regression

**Fig. 6:**
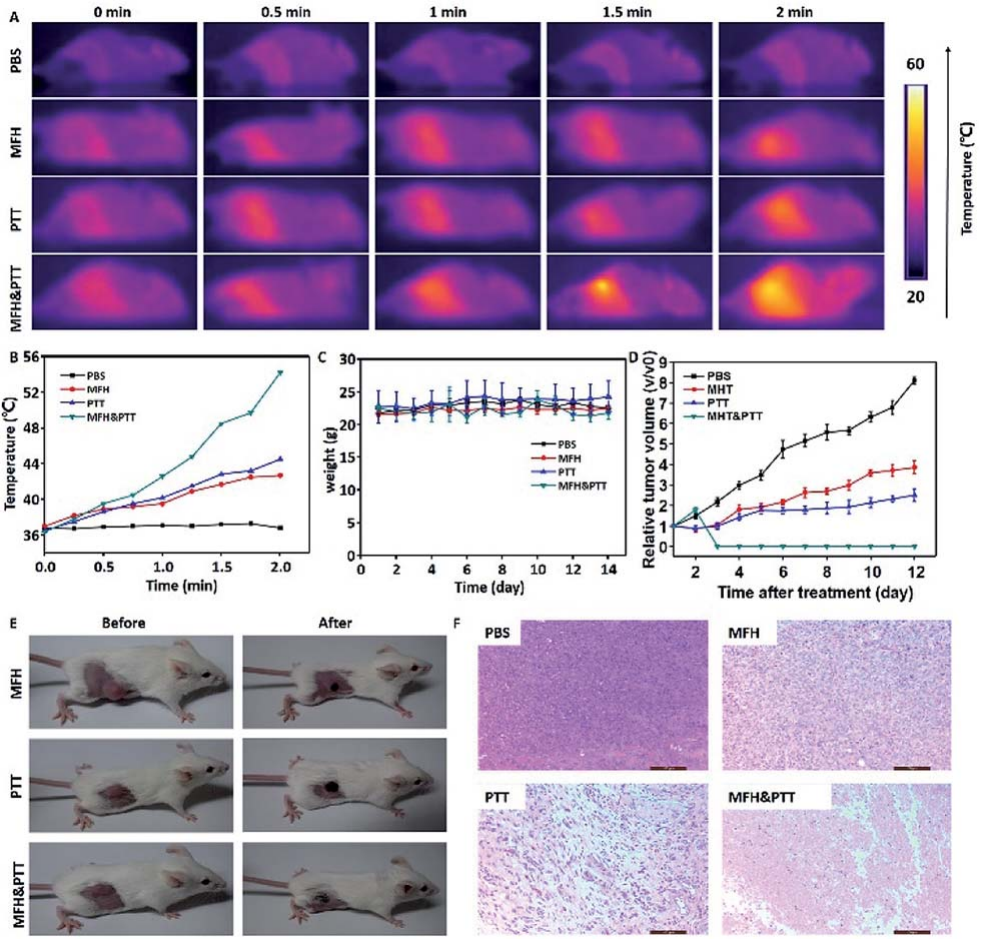
*In vivo* combination therapy of MPB NPs on 4T1 tumor-bearing mice. (A) Thermographic images of mice treated with an AC magnetic field (500 kHz, 12 kA m^−1^) and NIR laser (808 nm, 1.0 W cm^−2^) after injected the samples. (B) Temperature changes of tumor regions. (C) Change in body weight during therapy. (D) Relative tumor volume of the mice after different treatments. (E) Representative photos of 4T1 tumor-bearing mice after different treatments. (F) H&E staining images of tumor sections.

**Fig. 7:**
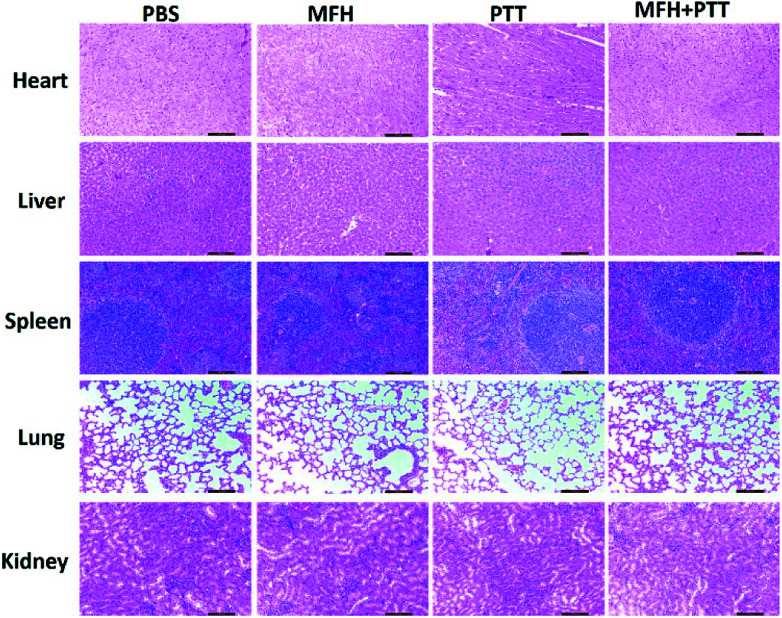
H&E stained images of heart, liver, spleen, lung, and kidney collected from mice of various groups with different treatments after 15 days. Scale bar, 100 μm.

Then the *in vivo* hyperthermia and photothermal combination therapy were carried out on the 4T1 tumor-bearing mice. As shown in the Fig. 6A, upon NIR-laser irradiation and magnetic hyperthermia, the tumor temperature did not show significant change for mice injected with PBS, and the final temperature of tumor site with single treatment was lower than dual-modal treatment. The corresponding temperature change of tumor was demonstrated in Fig. 6B. We could find that the temperature of the tumor with single treatment did not reached up to 44 °C, in contrast, the temperature of the tumor treated with dual treatment increased rapidly and maintained at about 54 °C, which was hot enough to kill cancer cells. In the following two weeks, the body weight was monitored every two days, as shown in Fig. 6C, no significant body weight change was observed in all groups showing that the MPB NPs had no significant side effect on the treated mice. The change in tumor size of the mice is shown in (Fig. 6D and E), the group treated with PBS showed rapid tumor growth, particularly, in the combination therapy group, the tumors were completely eradicated without regrowth or recurrence. A hematoxylin and eosin (H&E) staining analysis of tumors was also performed to further determine the therapeutic effect of different groups (Fig. 6F). The tumors treated with combination therapy showed much higher damage than those in the other groups. Meanwhile, as shown in the Fig. 7, the H&E images of the major organs from the 4T1 tumor-bearing mice in all groups showed no visible morphological changes caused by the MHT, PTT or the dual-modal treatment compared with the PBS group. These results further confirmed that the heat-generating MPB NPs under synergistic AC magnet and NIR could heat up and destroy the cancer cells without damaging the healthy ones. Finally, we could find that the MPB NPs with excellent biocompatibility could be applied as a nanotheranostic agent for the future imaging-guided PTT&MHT cancer treatment.

## 4. Conclusions

In summary, we have successfully designed and fabricated a multifunctional porous MnFe_2_O_4_/PB nanotheranostic agent for application in MRI-guided PTT and MHT. The as synthesized MPB NPs not only show outstanding MR contrast capabilities, but also exhibit excellent biocompatibility, high photothermal and magnetic hyperthermia conversion efficiency. Importantly, by means of introducing the surface coating of porous MnFe_2_O_4_ shell, it helps entrap large quantities of water around NPs and allow more efficient water exchange, leading to greatly improved MR contrast signals from 112.11 to 123.46 mM^−1^ s^−1^. Besides, the porous shell helps the NIR absorbance of the core, and then the extremely enhanced thermal effect can be obtained under synergistic combination of PTT and MHT. The SAR of MPB nanoparticles reached unprecedented levels of up to 4800 W g^−1^, compared with the SAR 1182 W g^−1^ of PTT under an 808 nm laser and 180 W g^−1^ of MHT under an external AC magnetic field. The *T*_1_–*T*_2_ dual-modal MRI-guided synergistic combination of photothermal and magnetic hyperthermia therapy exhibited the result in complete solid tumor ablation *in vivo*. These results demonstrate that the multifunctional MPB nanocomposites can be used as a powerful theranostic agents for magnetic resonance imaging-guided PTT and MHT, and open a new therapeutic window for the treatment of cancer and exhibit a huge potential in the biomedical field.

## Conflicts of interest

There are no conflicts to declare.

## Supplementary Material

RA-008-C8RA02946F-s001

## References

[cit1] Guo Z., Zhu S., Yong Y., Zhang X., Dong X. H., Du J. F., Xie J. N., Wang Q., Gu Z. J., Zhao Y. L. (2017). Adv. Mater..

[cit2] Wang Z., Shao D., Chang Z., Lu M., Wang Y., Yue J., Yang D., Li M., Xu Q., Dong W.-F. (2017). ACS Nano.

[cit3] Gao Q., Xie W. S., Wang Y., Wang D., Guo Z. H., Gao F., Zhao L. Y., Cai Q. (2018). RSC Adv..

[cit4] Huang C. Y., Qin H., Qian J., Zhang J. Y., Zhao S. Y., Changyi Y. Z., Li B. B., Zhang J. P., Zhu J. H., Xing D., Yang S. H., Li C. (2015). RSC Adv..

[cit5] Yang H., Xu M., Li S., Shen X., Li T. T., Yan J., Zhang C. C., Wu C. H., Zeng H. J., Liu Y. Y. (2016). RSC Adv..

[cit6] Cao Z. Y., Feng L. Z., Zhang G. B., Wang J. X., Shen S., Li D. D., Yang X. Z. (2018). Biomaterials.

[cit7] Huang X. J., Zhang W. L., Guan G. Q., Song G. S., Zou R. J., Hu J. Q. (2017). Acc. Chem. Res..

[cit8] Li X., Xing L. X., Zheng K. L., Wei P., Du L. F., Shen M. W., Shi X. Y. (2017). ACS Appl. Mater. Interfaces.

[cit9] Espinosa A., Di Corato R., Kolosnjaj-Tabi J., Flaud P., Pellegrino T., Wilhelm C. (2016). ACS Nano.

[cit10] Li J. B., Qu Y., Ren J., Yuan W. Z., Shi D. L. (2012). Nanotechnology.

[cit11] Liu Y., Lv X. L., Liu H., Zhou Z. J., Huang J. P., Lei S. L., Cai S. H., Chen Z., Guo Y. L., Chen Z. W., Zhou X., Nie L. M. (2018). Nanoscale.

[cit12] Zhou Z. J., Huang D. T., Bao J. F., Chen Q. L., Liu G., Chen Z., Chen X. Y., Gao J. H. (2012). Adv. Mater..

[cit13] Wang L. J., Wu Q., Tang S., Zeng J. F., Qiao R. R., Zhao P., Zhang Y., Hu F. Q., Gao M. Y. (2013). RSC Adv..

[cit14] Yang Y., Liu J., Liang C., Feng L., Fu T., Dong Z., Chao Y., Li Y., Lu G., Chen M., Liu Z. (2016). ACS Nano.

[cit15] Wang S., Lin J., Wang Z., Zhou Z., Bai R., Lu N., Liu Y., Fu X., Jacobson O., Fan W., Qu J., Chen S., Wang T., Huang P., Chen X. (2017). Adv. Mater..

[cit16] Kang N., Liu Y., Zhou Y., Wang D., Chen C., Ye S., Nie L., Ren L. (2016). Adv. Healthcare Mater..

[cit17] Cai H., Li K., Li J., Wen S., Chen Q., Shen M., Zheng L., Zhang G., Shi X. (2015). Small.

[cit18] Wang J., Zhou Z., Wang L., Wei J., Yang H., Yang S., Zhao J. (2015). RSC Adv..

[cit19] Das R., Rinaldi-Montes N., Alonso J., Amghouz Z., Garaio E., Garcia J. A., Gorria P., Blanco J. A., Phan M. H., Srikanth H. (2016). ACS Appl. Mater. Interfaces.

[cit20] Huang J., Xie J., Chen K., Bu L., Lee S., Cheng Z., Li X., Chen X. (2010). Chem. Commun..

[cit21] Cheng Y., Zhang S., Kang N., Huang J., Lv X., Wen K., Ye S., Chen Z., Zhou X., Ren L. (2017). ACS Appl. Mater. Interfaces.

[cit22] Cheng L., Gong H., Zhu W. W., Liu J. J., Wang X. Y., Liu G., Liu Z. (2014). Biomaterials.

[cit23] Zhu W. W., Liu K., Sun X. Q., Wang X., Li Y. G., Cheng L., Liu Z. (2015). ACS Appl. Mater. Interfaces.

[cit24] Cai X., Gao W., Ma M., Wu M., Zhang L., Zheng Y., Chen H., Shi J. (2015). Adv. Mater..

[cit25] Shokouhimehr M., Soehnlen E. S., Hao J., Griswold M., Flask C., Fan X., Basilion J. P., Basu S., Huang S. D. (2010). J. Mater. Chem..

[cit26] Bousseksou A., Molnar G., Salmon L., Nicolazzi W. (2011). Chem. Soc. Rev..

[cit27] Andreas K., Georgieva R., Ladwig M., Mueller S., Notter M., Sittinger M., Ringe J. (2012). Biomaterials.

[cit28] Fu G., Liu W., Feng S., Yue X. (2012). Chem. Commun..

[cit29] Liu Y., Guo Q., Zhu X., Feng W., Wang L., Ma L., Zhang G., Zhou J., Li F. (2016). Adv. Funct. Mater..

[cit30] Seo W. S., Lee J. H., Sun X., Suzuki Y., Mann D., Liu Z., Terashima M., Yang P. C., McConnell M. V., Nishimura D. G., Dai H. (2006). Nat. Mater..

[cit31] Zhang Q., Yin T., Gao G., Shapter J. G., Lai W., Huang P., Qi W., Song J., Cui D. (2017). ACS Appl. Mater. Interfaces.

[cit32] Deng K., Chen Y., Li C., Deng X., Hou Z., Cheng Z., Han Y., Xing B., Lin J. (2017). J. Mater. Chem. B.

[cit33] Shokouhimehr M., Soehnlen E. S., Khitrin A., Basu S., Huang S. D. (2010). Inorg. Chem. Commun..

[cit34] Shah S. A., Majeed A., Rashid K., Awan S.-U. (2013). Mater. Chem. Phys..

[cit35] Ma M., Wu Y., Zhou H., Sun Y. K., Zhang Y., Gu N. (2004). J. Magn. Magn. Mater..

[cit36] Liu Y., Kang N., Lv J., Zhou Z., Zhao Q., Ma L., Chen Z., Ren L., Nie L. (2016). Adv. Mater..

[cit37] Chen G., Ma B., Wang Y., Xie R., Li C., Dou K., Gong S. (2017). ACS Appl. Mater. Interfaces.

[cit38] Ren S., Cheng X., Chen M., Liu C., Zhao P., Huang W., He J., Zhou Z., Miao L. (2017). ACS Appl. Mater. Interfaces.

[cit39] Miao Z.-H., Wang H., Yang H., Li Z.-L., Zhen L., Xu C.-Y. (2015). ACS Appl. Mater. Interfaces.

[cit40] Shanmugam V., Selvakumar S., Yeh C.-S. (2014). Chem. Soc. Rev..

[cit41] Huang G., Zhang K.-L., Chen S., Li S.-H., Wang L.-L., Wang L.-P., Liu R., Gao J., Yang H.-H. (2017). J. Mater. Chem. B.

