# Supplementary material for: Porous MnFe2O4-decorated PB nanocomposites: a new theranostic agent for boosted T1/T2 MRI-guided synergistic photothermal/magnetic hyperthermia
Source: RSC Adv. 2018 May 22;8(33):18647–55. doi: 10.1039/c8ra02946f (PMC9080558; doi:10.1039/c8ra02946f)
Supplement: RA-008-C8RA02946F-s001 [file RA-008-C8RA02946F-s001.pdf]

Electronic Supplementary Material (ESI) for RSC Advances.
This journal is © The Royal Society of Chemistry 2018

# Supporting Information

# Porous $MnFe_2O_4$-Decorated PB Nanocomposites: a New Theranostic Agent for Boosted $T_1/T_2$ MRI-Guided Synergistic Photothermal/Magnetic Hyperthermia

Xi Zhou,[a] Xiaolin Lv,[a] Wen Zhao,[a] Tiantian Zhou,[b] Shupeng Zhang,[a] Zhan Shi,[c] Shefang Ye*,[a] Lei Ren,[a] and Zhiwei Chen*,[b]

[a]Key Laboratory of Biomedical Engineering of Fujian Province University/Research Center of Biomedical Engineering Technology of Xiamen, Department of Biomaterials, College of Materials, Xiamen University, Xiamen 361005, P. R. China, [b]Department of Electronic Science, Fujian Provincial Key Laboratory of Plasma and Magnetic Resonance Research, Xiamen University, Xiamen 361005, P. R. China, and [c]Department of Materials Science and Engineering, College of Materials, Xiamen University, Xiamen 361005, P. R. China.

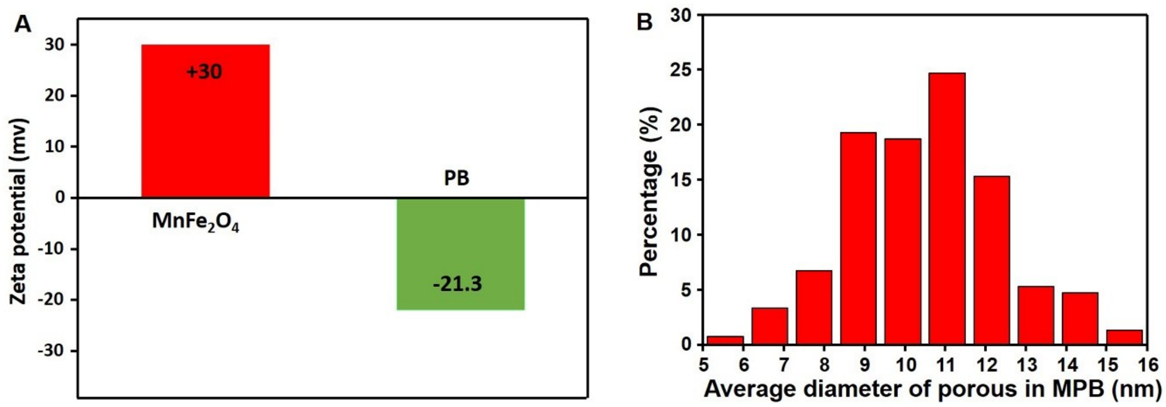


**Fig. S1** (A) The zeta potentials of $MnFe_2O_4$ and PB in water. (B) The average diameter of porous in MPB NPs was measured in SEM images by Nano Measurer.

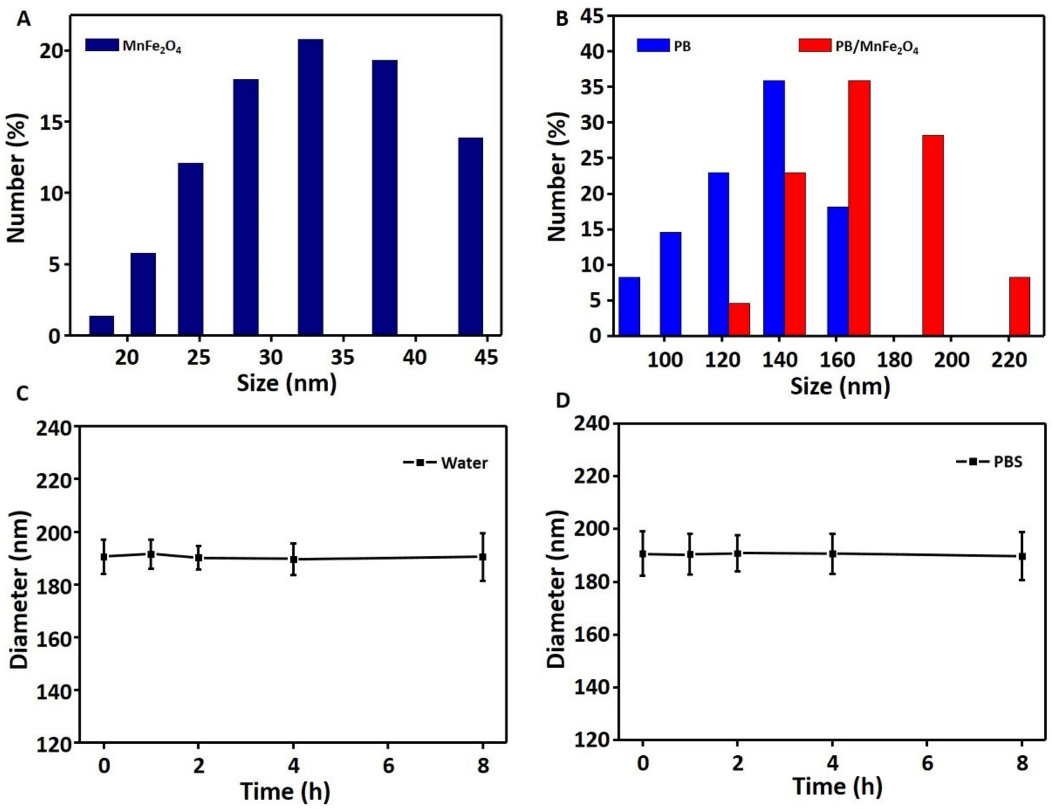


**Fig. S2** Hydrodynamic particle size of the $MnFe_2O_4$ (A), PB and MPB (B) NPs in water; The diameter of MPB NPs in water (C) and PBS (D) at different time intervals.

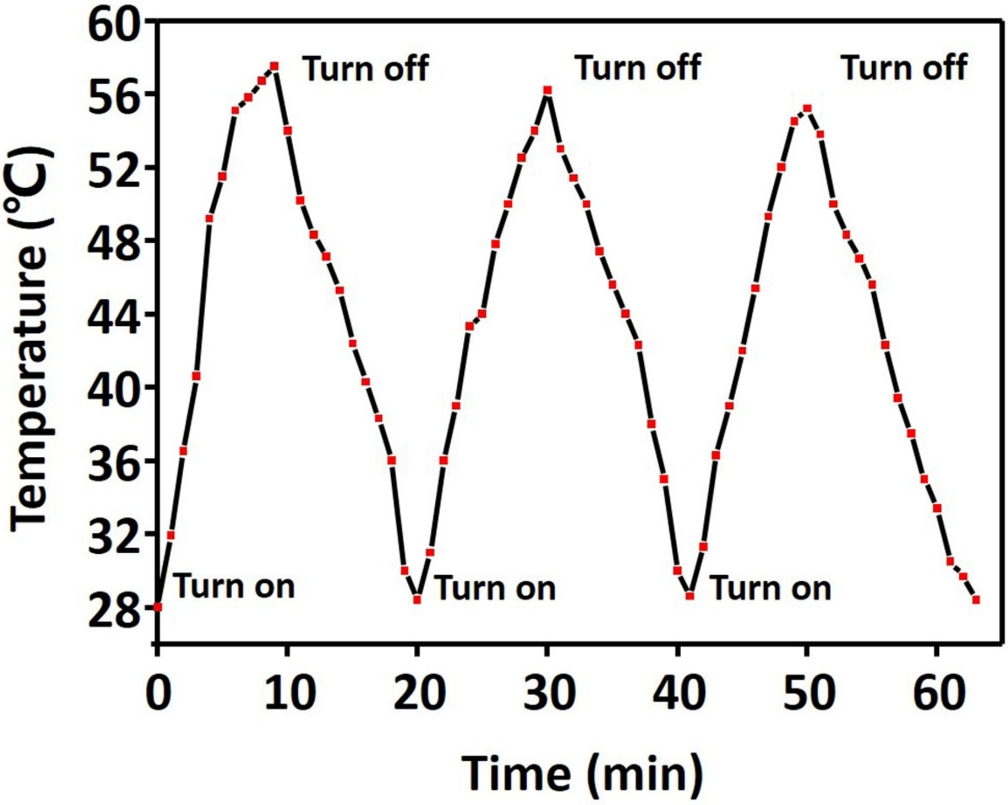


**Fig. S3** Temperature variations of the MPB NPs under irradiation of an 808 nm laser (1.0 W $cm^{-2}$) and AC magnetic field (20A) synergistically for three cycles.

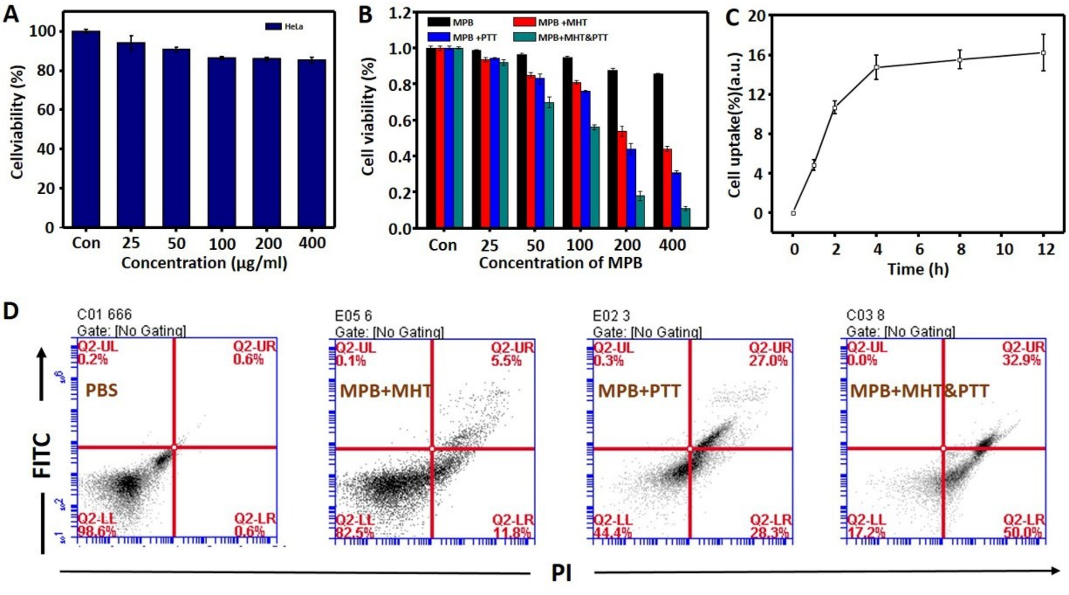


**Fig. S4** *In vitro* experiments. (A) *In vitro* cytotoxicity of MPB NPs against Hela after 24 h incubation. (B)

Cell viability of Hela cells incubated with MPB, MPB+PTT, MPB+MHT and MPB+MHT&PTT. (C) Cellular uptake of MPB NPs at different time intervals with Hela cells. (D) Flow cytometry analysis the toxicities of PBS, MPB+MHT, MPB+PTT and MPB+MHT&PTT with Hela cells.

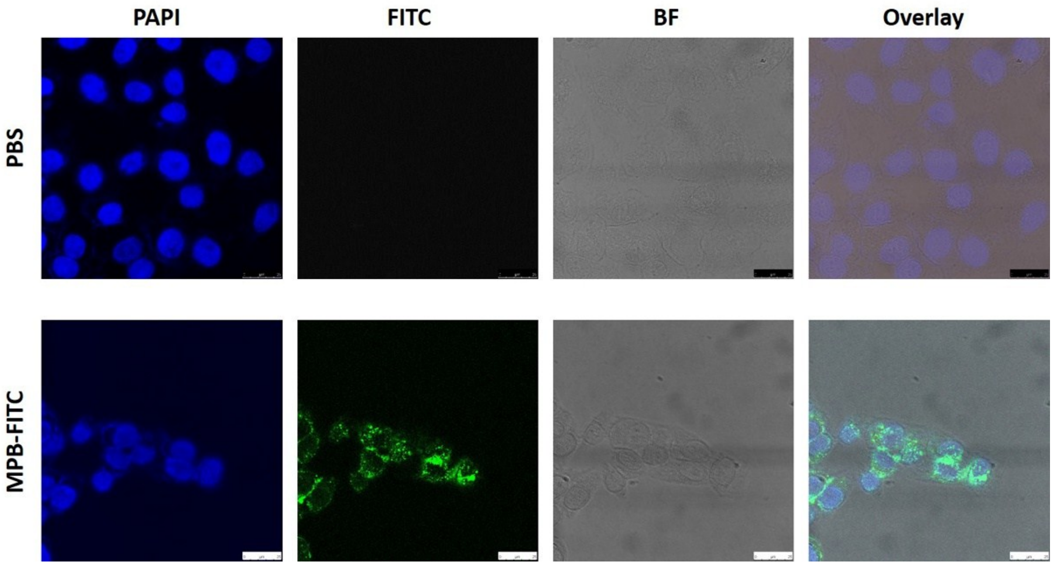


**Fig. S5** CLSM images of Hela cells incubated with PBS and MPB-FITC for 4h. The cells were stained by DAPI (blue) and FITC was green.
